# A novel CSF-1R mutation in a family with hereditary diffuse leukoencephalopathy with axonal spheroids misdiagnosed as hydrocephalus

**DOI:** 10.1007/s10048-019-00579-0

**Published:** 2019-05-16

**Authors:** Miaomiao Wang, Xinqing Zhang

**Affiliations:** Department of Neurology, Xuanwu Hospital of Capital Medical University, Beijing, 100053 China

**Keywords:** Hereditary diffuse leukoencephalopathy with axonal spheroids (HDLS), Novel mutation, Hydrocephalus, Gait disturbance

## Abstract

Hereditary diffuse leukoencephalopathy with axonal spheroids (HDLS) is a rare autosomal dominant disease caused by mutations in the colony stimulating factor 1 receptor (CSF1R) gene that often results in cognitive impairment, psychiatric disorders, motor dysfunction and seizure. We report familial cases of a novel CSF1R mutation causing HDLS similar to hydrocephalus. The patients initially presented with a gait disturbance and then developed progressive cognitive decline, urinary incontinence, epileptic seizures and became bedridden as the disease progressed. A brain magnetic resonance imaging (MRI) scan revealed striking ventricular enlargement and diffuse brain atrophy with frontotemporal predominance, which was later accompanied by white matter changes. Genetic testing in this family showed a novel c.2552T>C (p.L851P) mutation in exon 19 of the CSF1R gene. However, three gene carriers in the family remained clinically asymptomatic. Because of its heterogeneous clinical phenotypes, HDLS patients are often misdiagnosed with other diseases. This is the first genetically proven HDLS case resembling hydrocephalus, and the clinical symptoms of HDLS may be related to the specific genetic mutation.

## Introduction

Hereditary diffuse leukoencephalopathy with axonal spheroids (HDLS) is a progressive neurodegenerative disease first described and pathologically defined in a large Swedish family in 1984 by Axelsson R et al. [1]. Typically, HDLS results in personality changes and is often associated with cognitive impairment, motor dysfunction and other clinical presentations, such as stroke-like episodes, sensory disturbance, dizziness, fatigue and epilepsy [2]. Spotty calcification detected on computer tomography (CT) in the frontal pericallosal regions has been reported as a specific finding in patients with HDLS [3] and brain magnetic resonance imaging (MRI) shows asymmetric deep white matter lesions with frontoparietal predominance, followed by brain atrophy [4, 5]. Since HDLS was identified as a rare autosomal dominant disease caused by a mutation of the colony stimulating factor 1 receptor (CSF1R) gene located on chromosome 5 (5q32) [6], more than 70 pathogenic mutations have been reported in both hereditary and sporadic cases [7]. Due to the significant variability in genotypes and phenotypes, patients with HDLS have often been misdiagnosed with other diseases [8–12].

Here, we report a new HDLS family with a novel CSF1R mutation, in which the prominent clinical characteristics are associated with hydrocephalus.

## Case report

**Fig. 1:**
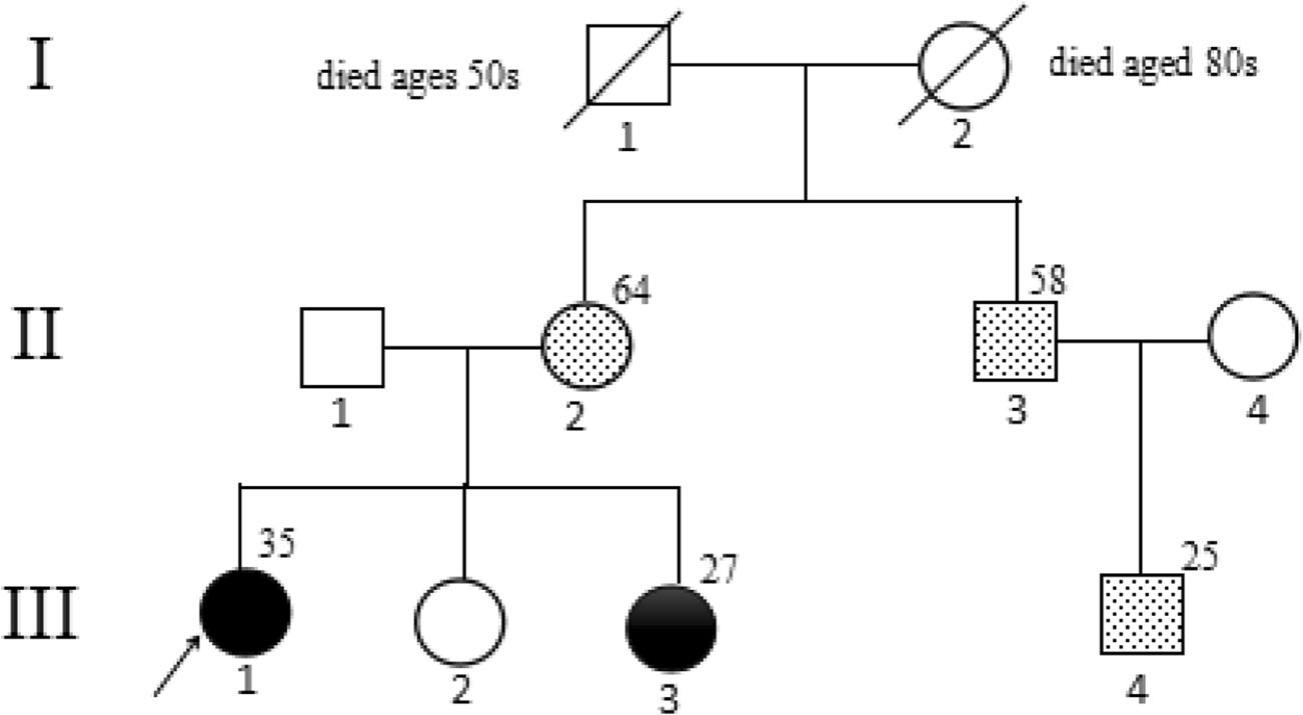
Family tree. A three-generation pedigree of the family with genotyping c.2552T>C (p.L851P) of the mutation in the CSF-1R gene is shown. Squares represent men and circles represent women. Black solid symbols indicate affected individuals. Hashed filled symbols indicate asymptomatic carriers. White symbols indicate healthy individuals. An arrow indicates the proband. Deceased individual is marked by a slash

This ten-member Chinese family was recruited for this report (shown in Fig. 1—family tree). We examined the clinical and imaging features of two patients (III-1 and III-3) from this family. The maternal grandfather of the patients (I-1) died of cerebrovascular disease in his 50s, and their maternal grandmother (II-2) was reported to be healthy in her 80s. Neither of their maternal grandparents had undergone genetic testing. The mother (II-2) and other two members carried the same CSF1R mutation but remained asymptomatic at the ages of 64, 58 and 30, respectively. In contrast, the father (II-1) and sister (III-2) of the patients were clinically unaffected and had normal genes.

## Patient III-1

**Fig. 2:**
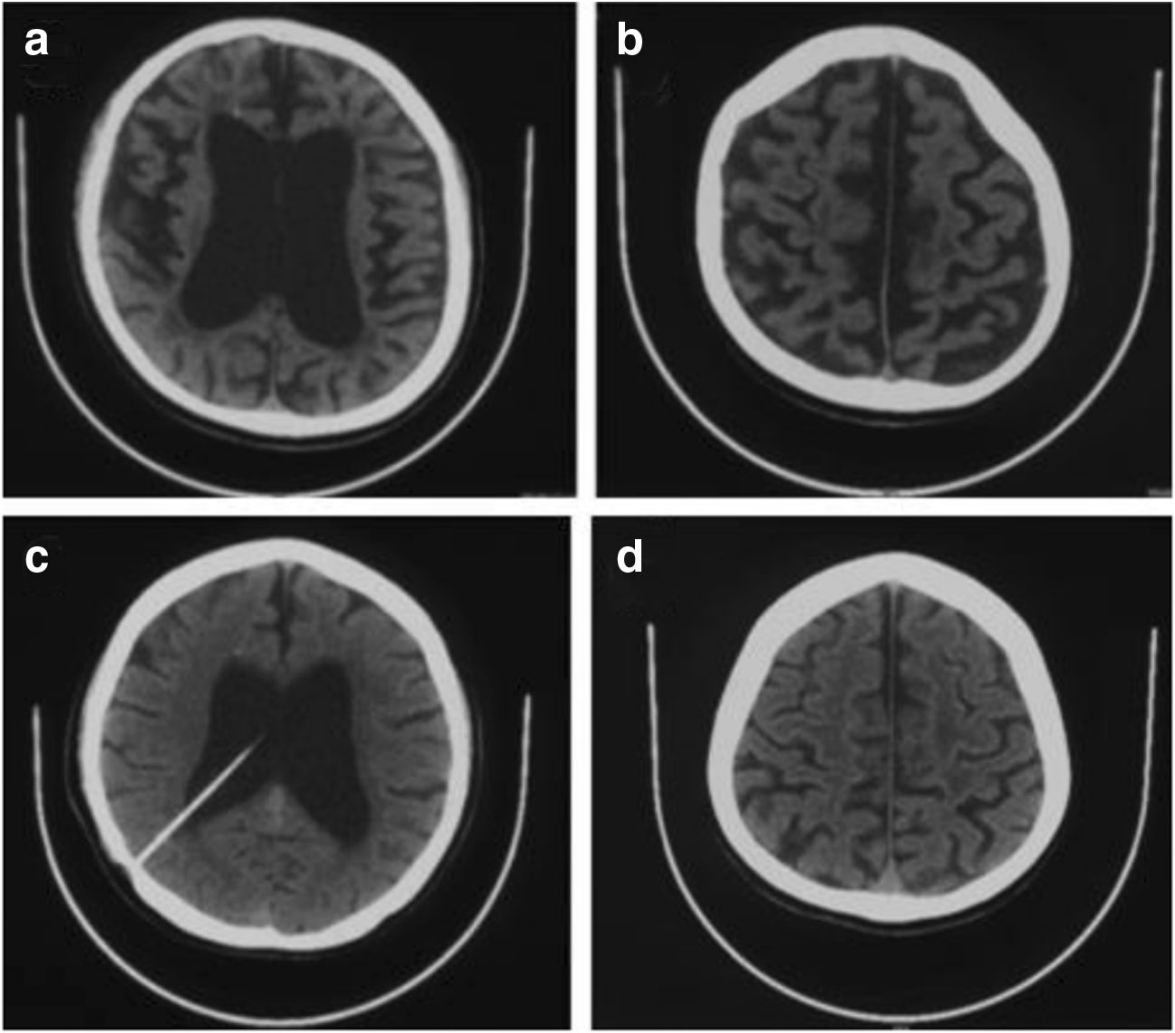
CT. CT scans show diffuse brain atrophy and enlargement of the ventricles with a small calcification located near the anterior horns of the right lateral ventricles. **a**, **b** Neuroimaging of patient III-1. **c**, **d** Neuroimaging of patient III-3

This 35-year-old female patient was the proband. At age 28, she developed an abnormal right leg posture when walking. At age 29, she displayed right leg spasms, weakness of the right arm and slurred speech. At age 30, the patient’s condition continued to worsen. She experienced stiffness in all four limbs and required assistance when standing and walking. At approximately the same time, her father noted a subtle change in her personality and behaviour, which was associated with irritability and seemingly unprovoked laughter. Brain CT showed a small calcification located near the anterior horns of the right lateral ventricles (shown in Fig. 2—CT). MRI revealed apparent diffuse atrophy of brain tissue and enlargement of the lateral and third ventricles. Then, the patient was diagnosed with hydrocephalus and underwent a cerebrospinal fluid tap test, but her symptoms continued to progress. Subsequently, she received allogenic haematopoietic stem cell transplantation (HSCT) treatment but with no effect.

**Fig. 3:**
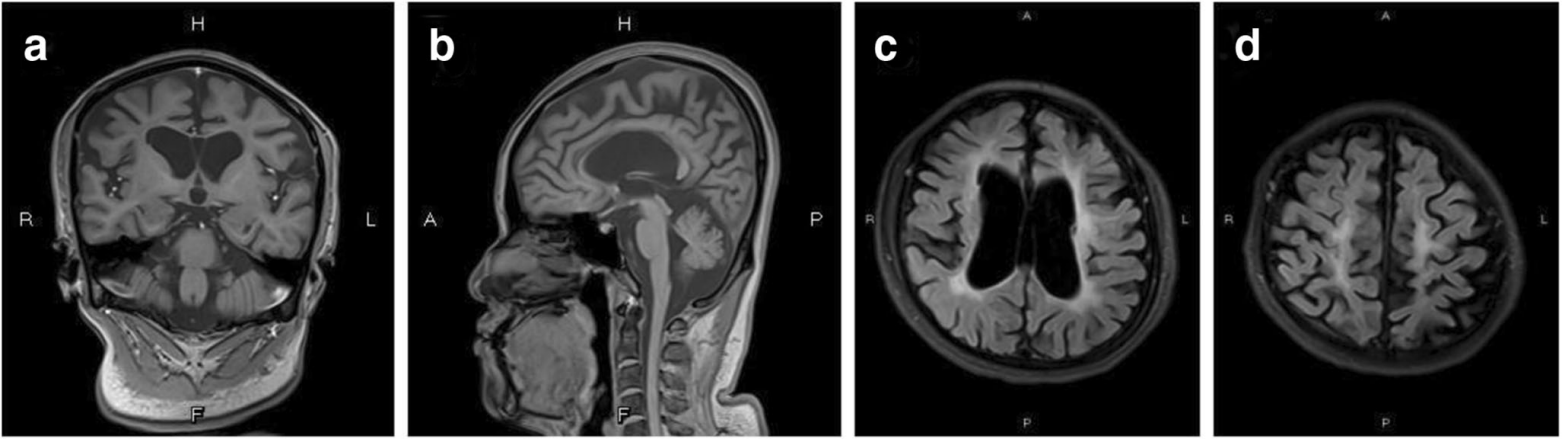
MRI. Neuroimaging of patient III-1. **a** T1-weighted coronal MRI shows diffuse brain atrophy with a frontotemporal predominance and enlargement of the ventricles. **b** T1-weighted sagittal MRI shows brain atrophy and severe thinning of the corpus callosum. **c** FLAIR axial MRI shows brain atrophy, ventriculomegaly and symmetrical hyperintensity of the periventricular white matter. **d** FLAIR axial MRI shows subcortical bifrontal and frontoparietal cerebral white matter hyperintensity

At the age of 31, the patient was admitted to our institution with severe walking difficulties, progressive memory loss, slurred speech and involuntary tremors in both upper limbs. On neurological examination, the patient’s cranial nerve appeared normal. She showed increased muscle tone in the extremities, although her muscle strength was normal. She also showed active deep tendon reflexes in all four limbs. The right foot displayed a positive Babinski sign, and the Babinski sign was suspected as positive in the left foot. Her Wechsler Memory Scale score was 19, and her memory quotient was 6. The Wechsler Adult Intelligence Scale suggested that the patient’s IQ was impaired. Both laboratory tests and routine cerebrospinal fluid (CSF) studies were normal. On MRI, in addition to diffuse brain atrophy, changes in white matter were detected. Diffuse brain atrophy with frontotemporal predominance, enlargement of ventricles and thinning of the corpus callosum on sagittal and coronal T1-weighted images (T1WI) were observed, along with abnormal signals in the deep white matter located in the periventricular areas and subfrontal parietal cortex, which were shown as hyperintensities on T2- weighted imaging (T2WI) and fluid-attenuated inversion recovery (FLAIR) sequences (shown in Fig. 3—MRI).

At age 32, the patient gradually became bedridden and unable to manage herself due to dysphagia, urinary and bowel incontinence, rigidity, apathy and dementia. She was finally diagnosed with HDLS, as confirmed by genetic analyses, which revealed a heterozygous mutation (c.2552T>C) in exon 19 of the CSF-1R gene on chromosome 5, resulting in an amino acid substitution of leucine (L) to proline (P) at codon position 851 (p.L851P).

## Patient III-3

This patient was a 27-year-old female and the youngest sister of patient III-1.

**Fig. 4:**
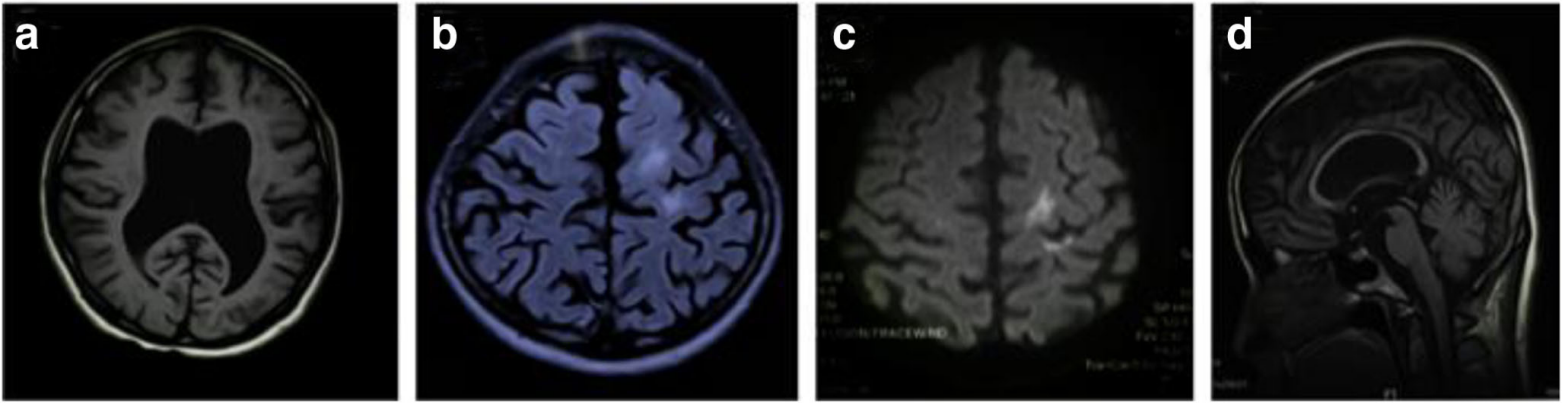
MRI. Neuroimaging of patient III-3. **a** T1-weighted axial MRI shows mild diffuse brain atrophy and enlargement of the ventricles. **b** FLAIR axial MRI shows asymmetrical hyperintensity with a frontoparietal cerebral white matter predominance. **c** Diffusion-weighted imaging (DWI) shows isolated spots of hyperintense signal in the left subcortical frontoparietal cerebral white matter hyperintensity. **d** T1-weighted sagittal MRI shows brain atrophy and mild thinning of the corpus callosum

The patient was first hospitalised at age 25 with a 1-year history of gradual progression of a gait disorder, which resulted in an increased likelihood falling. On examination, her Mini-Mental State Examination (MMSE) score was 25/30. On neurological examination, the patient showed mild weakness and increased muscle tone in the right limbs. Brain MRI revealed enlargement of the ventricles, which was associated with isolated spots of hyperintense on diffusion-weighted imaging (DWI) and a bright asymmetrical signal on FLAIR in the left frontoparietal lobe and left lateral ventricle (shown in Fig. 4—MRI). Based on the MRI results, the patient was initially diagnosed with multiple cerebral infarction and hydrocephalus. She was treated with circulation improvement and a ventriculoperitoneal shunt in a local hospital. However, her gait disorder continued to progress, and her cognitive function declined in the following year.

At age 26, the patient was admitted to our institution due to progressive gait disturbance and slight cognitive decline. Her MMSE score had decreased to 18/30. A neurological examination showed muscular hypertonia and ataxia of the right limbs. Hyperreflexia and ankle clonus were presented in the right leg. Because of the location of the ventriculoperitoneal shunt, subsequent MRI was not performed. Repeated CT scans showed diffuse cerebral atrophy with enlargement of the ventricles, and a small calcification located near the anterior horns of the right lateral ventricles is seen (shown in Fig. 2—CT). An ^18^ F-fluorodeoxyglucose positron emission tomography (FDG-PET) scan showed a reduction in glucose uptake in the cerebral lobes, especially the frontal lobes. DNA sequencing of the patient revealed genome revealed a heterozygous mutation (c.2552T>C) in exon 19 of the CSF-1R gene on chromosome 5, which is the same as the patient III-1.

At present, the patient is wheelchair-bound because of spasticity in the right limbs and has urinary and bowel incontinence and progressive cognitive impairment.

## Genetic analysis

Genomic DNA was extracted from peripheral blood leukocytes after obtaining informed consent. The fragments of limited exons and adjacent intron areas were amplified by polymerase chain reaction (PCR) and subjected to Sanger sequencing.

**Fig. 5:**
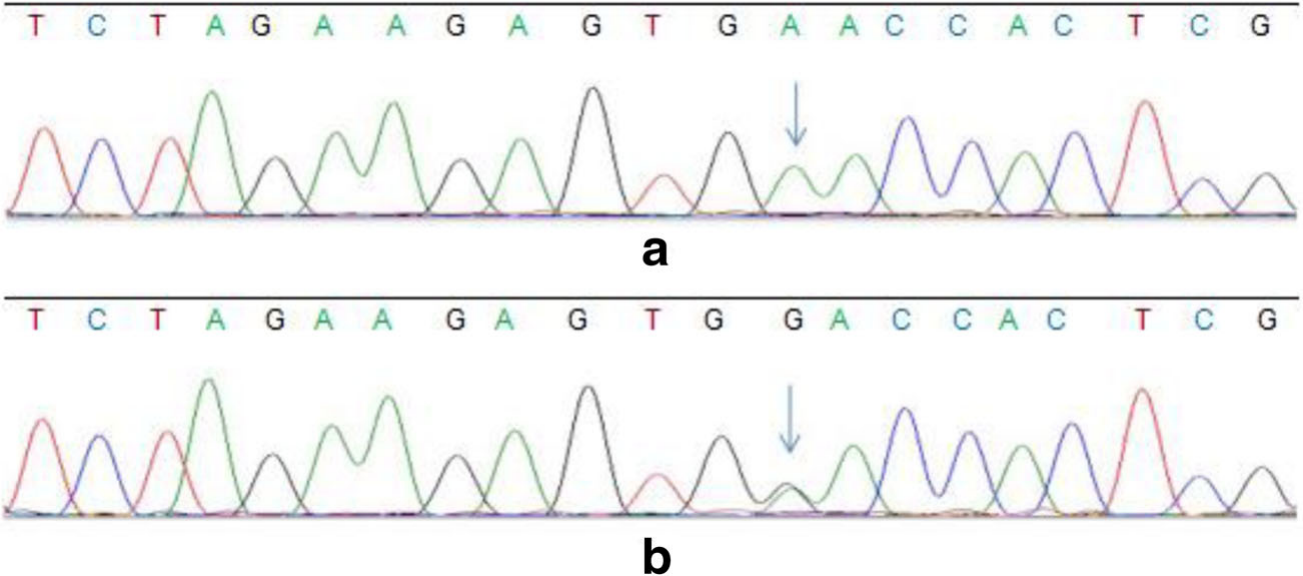
DNA sequencing findings**.** DNA sequencing of the patients’ father revealed no nucleotide substitution in the CSF1R gene (**a**). DNA sequencing of the patients revealed a heterozygous mutation (c.2552T>C) in exon 19 of the CSF-1R gene on chromosome 5, resulting in an amino acid substitution of leucine (L) to proline (P) at codon position 851 (p.L851P) (**b**)

A novel heterozygous c.2552T>C (p.L851P) mutation was revealed in exon 19 of the CSF1R gene, resulting in an amino acid substitution of leucine (L) to proline (P) at codon position 851 (p.L851P), which was predicted to be damaged by the SIFT algorithm. (shown in Fig. 5—DNA sequencing findings). This mutation has not been previously reported.

## Discussion

HDLS is a hereditary cerebral white matter degenerative disease caused by mutations of CSF1R and results in personality changes, followed by cognitive decline and motor impairment, particularly affecting the gait [13]. The family we report here is consistent with previous descriptions in some aspects. The onset ages of our patients were 28 and 25 years old, which is consistent with the typical onset age (ranging from 8 to 78 years old) [6, 10, 14]. Both patients developed motor dysfunction, cognitive decline and epileptic seizures in the final disease stage. Spotty calcification in the white matter was observed by CT scan which was considered a common manifestation in patients with HDLS [3, 15]. The final MRI changes were similar to those of classical HDLS, with asymmetrical lesions predominant in the anterior deep white matter along with thinning of the corpus callosum and brain atrophy [4, 5].

However, there are some unique features in our report.

The patients we reported initially presented with motor dysfunction, a symptom that often develops as the disease progresses. For most HDLS patients, the first clinical symptom is cognitive impairment or psychiatric disorder, including all six cases described in the Chinese mainland [16–18]. Stabile [2] reported that motor dysfunction had a high incidence rate as the first symptom among women in their 20s, who then developed seizures, dementia and other pyramidal dysfunctions as the disease progressed [19], which supports our findings.

Although the MRI results in the late phase of the disease were similar to previously reported neuroimaging findings, our patients’ first brain MRI results revealed markedly diffuse brain atrophy and enlargement of the lateral and third ventricles. Additionally, patient III-3 showed spotty bright DWI signals in the left frontoparietal lobe and left lateral ventricle, which led to an initial diagnosis of multiple lacunar cerebral infarctions. A previous report showed that strong DWI signals could be observed in the early stage of HDLS, but unlike cerebral infarctions, these abnormal signals would persist [20]. We hypothesised that this imaging manifestation might be related to the mutation of CSF1R gene. These types of noticeable imaging changes combined with the clinical manifestations of gait instability, cognitive impairment and urinary incontinence show a strong resemblance to hydrocephalus [21], thus leading to ineffective treatment.

The family in this study was found to have a novel CSF1R gene mutation c.2552T>C (p.L851P), which has not been previously reported worldwide. The gene carriers in the family were found to have different phenotypes. The patients were severely affected at 28 and 25 years old, whereas their mother, who carried the same CSF1R mutation remained healthy at 64 years, and their uncle (58 years) and cousin (30 years) also remained healthy. This pattern of asymptomatic mutation might represent a lack of penetrance or a late-onset phenotype that had not yet manifested, as previously reported in the literature [22]. Due to the broad age of onset and various clinical manifestations, it is not certain whether the asymptomatic carriers will develop symptoms with age. We will closely monitor this family to improve our understanding of the disease.

In terms of treatment, there are no effective drugs currently known to halt or alter the course of HDLS. Patient III-1 was treated with HSCT, but it did not improve any of her symptoms. A previous report described an affected individual who successfully underwent an allogenic bone marrow transplantation from her healthy sibling and maintained a stable condition for the following 15 years [23]. Thus, therapies need to be explored for HDLS patients.

## Conclusion

The c.2552T>C missense mutation identified in this family has not been previously reported. The clinical and neuroimaging features of the family reported here resembled hydrocephalus. HDLS was verified by genetic testing. In contrast to previous reports, the initial symptom of HDLS can be motor dysfunction. Brain atrophy may be the prominent finding on MRI. The clinical features of the family may be related to the novel mutation of p.L851P. HDLS should be considered a differential diagnosis when a patient presents with the clinical characteristics as mentioned above.
